# Decoding Synaptic Diversity: Molecular Architectures, Phase Transitions, and Shared Postsynaptic Failure in Alzheimer’s and Parkinson’s Disease

**DOI:** 10.3390/cells15161433

**Published:** 2026-08-09

**Authors:** Giovanni Luca Cipriano, Ivan Anchesi, Alessia Floramo, Veronica Argento, Sara Spinelli, Maria Francesca Astorino, Marco Calabrò, Osvaldo Artimagnella

**Affiliations:** 1 Centro Neurolesi “Bonino-Pulejo”, IRCCS, Via Provinciale Palermo, Contrada Casazza, 98124 Messina, Italy; giovanniluca.cipriano@irccsme.it (G.L.C.); ivan.anchesi@irccsme.it (I.A.); alessia.floramo@irccsme.it (A.F.); veronica.argento@irccsme.it (V.A.);; 2 Department of Biomedical and Dental Sciences and Morpho-Functional Imaging—BIOMORF, University of Messina, 98125 Messina, Italy; mastorino@unime.it (M.F.A.); mcalabro@unime.it (M.C.)

**Keywords:** synaptome, subsynaptic domains, liquid–liquid phase separation, Alzheimer’s disease, Parkinson’s disease, precision synaptopharmacology, nanocolumns, tetrapartite synapse, neuroinflammation, neurorehabilitation

## Abstract

Synaptic failure is the most accurate pathological correlate of cognitive and motor decline in neurodegenerative diseases. However, the molecular logic governing selective synaptic vulnerability in Alzheimer’s (AD) and Parkinson’s (PD) remains a fundamental enigma. This review dissects the hierarchical organization of the synaptome, arguing that synaptic decay is not a generic process of attrition but a specific collapse of subsynaptic domains (SSDs) and trans-synaptic nanocolumns, considered here within the framework of the tetrapartite synapse, which comprises the presynaptic and postsynaptic compartments together with glia and the perisynaptic extracellular matrix. We use the term pathological convergence in a restricted sense, to denote that, although the primary aggregates differ, the two diseases converge on the same postsynaptic scaffolding hubs and on a comparable loss of condensate fluidity. We propose a biophysical model where the Post-Synaptic Density (PSD) matrix, governed by liquid–liquid phase separation (LLPS), may undergo a pathological liquid-to-solid transition—characterized by condensate maturation and the formation of insoluble protein aggregates—driven by proteotoxic species. Specifically, we analyze how Aβ-mediated zinc sequestration disrupts the Shank-SAM scaffold hierarchy in AD, while α-synuclein aggregates arrest presynaptic vesicle dynamics and mitochondrial homeostasis in PD. Furthermore, we explore the emerging frontier of “Precision Synaptopharmacology,” highlighting how targeted modulation of protein–protein interaction (PPIs), synthetic synaptic organizers (e.g., CPTX), and phase-stabilizing chaperones can restore nanocolumn alignment and synaptic fluidity. We also set out the principal limitations of these strategies, including blood–brain barrier delivery, off-target effects, the immaturity of condensate-directed pharmacology and the incomplete translation of rodent findings to human disease, and we consider the vascular and peripheral contributions that modify the synaptic environment. By integrating recent advances in super-resolution microscopy, systems biology, and activity-based neurorehabilitation, we provide a comprehensive framework for shifting neuroprotective strategies toward the precision engineering and functional recovery of synaptic nano-architecture.

## 1. Introduction: The Concept of Synaptic Diversity

### 1.1. Defining the “Synaptome”: Beyond the Generic Synapse Model

The paradigm of molecular neurobiology has progressively shifted from a reductionist genomic focus, identifying isolated disease-associated risk loci, to a comprehensive systems biology framework [1]. In this modern context, the classical connectionist model of a generic, monolithic synapse is now integrated into a multi-scale systems biology framework [2]. It is now understood that synapses contain thousands of distinct proteins with highly complex cell biological and signaling properties, giving rise to the concept of the “synaptome”, the complete, brain-wide catalogue of synapses characterized by their vast molecular diversity, nanoarchitecture, and protein turnover rates [3].

To systematically classify this complexity, the expert-curated, evidence-based database SynGO was established, defining 87 distinct synaptic locations and 179 synaptic processes. Data from SynGO reveal that synaptic genes are exceptionally well-conserved across evolution and display a significantly lower tolerance to loss-of-function mutations compared to non-synaptic genes [4]. The transition from the genome to this functional synaptome is heavily dictated by complex protein–protein interaction (PPI) networks and dynamic post-translational modifications (PTMs) [5,6]. Interrogation of curated databases demonstrates that synaptic proteins exhibit a significantly higher incidence of critical PTMs, such as phosphorylation, ubiquitination, and palmitoylation, than their non-synaptic counterparts [7]. These modifications generate a vast array of unique “proteoforms” from a single genomic sequence, which dynamically regulate the stability of postsynaptic scaffolding domains, coordinate liquid–liquid phase separation (LLPS), and dictate the temporal properties of synaptic transmission [8]. Pathological disruptions that alter these specific PTMs and PPI networks directly compromise synaptic proteostasis, ultimately driving the selective vulnerability seen in neurodegenerative synaptopathies [9].

### 1.2. Spatial and Cell-Type-Specific Protein Distribution Across Brain Regions

The molecular composition of the synaptome is not homogeneously distributed; rather, it is highly compartmentalized across distinct brain regions and strictly tailored to specific neuronal cell types [10]. High-dimensional proteomic profiling, utilizing techniques such as Fluorescence-Activated Synaptosome Sorting (FASS), has isolated the proteomes of genetically defined synaptic populations, revealing that excitatory and inhibitory synapses harbor fundamentally distinct, mutually exclusive protein communities [10].

The molecular characterization of GABAergic cells perfectly illustrates the extreme specificity of synaptic diversity [11]. Deep proteomic mapping demonstrates that different classes of inhibitory interneurons, specifically parvalbumin (PV+), somatostatin (SST+), and vasoactive intestinal peptide (VIP+) expressing cells, utilize entirely distinct synaptic proteomes [7,12]. For instance, to sustain their high-frequency firing rates, PV+ synapses are selectively enriched in specific mitochondrial proteins, hyperpolarization-activated cyclic nucleotide-gated (HCN) channels, and voltage-gated potassium channels (e.g., Kcnc1-3/Kv3.1-3), alongside specialized presynaptic vesicle sensors like Synaptotagmin-2 (Syt2) [13,14]. Conversely, the VIP+ synaptic proteome is distinctly characterized by the enrichment of neuromodulatory G-protein coupled receptors, including cannabinoid receptor 1 (Cnr1) [15] and metabotropic glutamate receptors (Grm1 and Grm7) [16].

Crucially, the assembly and stability of these diverse synaptic populations are governed by the differential expression of cell adhesion molecules (CAMs), which act as a trans-synaptic molecular “barcode” to dictate the precise logic of neural circuit wiring [17]. CAMs such as Neurexins, Neuroligins, and Leucine-Rich Repeat Transmembrane proteins (LRRTMs) are highly enriched in a cell-type-specific manner. For example, the Neurexin (Nrxn) family utilizes extensive alternative splicing to create diverse isoforms that bind to specific postsynaptic ligands [18]. The splicing of specific segments (such as AS4 and AS5 in Nrxn3) is strictly regulated in PV+, SST+, and VIP+ interneurons by the cell-type-specific expression of STAR family RNA-binding proteins, including SAM68, SLM1, and SLM2 [19,20]. By regulating these trans-synaptic PPIs, CAMs ensure the structural alignment of the pre- and postsynaptic compartments, establishing the stable molecular architectures required to maintain functional diversity across the brain [21].

### 1.3. The Tetrapartite Synapse: Glia and the Extracellular Matrix as Constitutive Synaptic Elements

The compartments described above do not operate in isolation. Contemporary models describe the synapse as a tetrapartite structure, in which the presynaptic terminal and the postsynaptic compartment are complemented by perisynaptic glial processes and by the perisynaptic extracellular matrix (ECM) [22,23,24]. Astrocytic leaflets regulate transmitter clearance and ion buffering, while microglial processes contact synapses under physiological conditions and participate in their surveillance and remodeling [25]. The fourth element, the ECM, comprises the diffuse perisynaptic matrix and the condensed perineuronal nets (PNNs) that enwrap subsets of neurons, most notably parvalbumin-positive interneurons, and constrain the lateral mobility of receptors and adhesion molecules [22]. Glial cells are themselves the principal remodellers of this matrix, secreting and degrading its components through matrix metalloproteinases, so that a change in glial state is translated directly into a change in synaptic stability [22,25].

Framing synaptic diversity in these terms has two consequences that recur throughout this review. First, neuroinflammation is not a late, downstream event. Complement proteins of the classical cascade, C1q in particular, are constitutive components of the synaptic cleft and participate in developmental pruning, and their reactivation is detectable at early disease stages, before overt neuronal loss [24,26]. Glia-derived signals therefore act on synaptic architecture from the outset, and are treated here both as a physiological determinant of synaptic identity (Section 1 and Section 3) and as an effector of selective vulnerability (Section 4 and Section 5). Second, because the ECM and the glial compartments are themselves region- and cell-type-specific, they contribute an additional layer of molecular heterogeneity to the synaptome, beyond the neuronal proteome described above [22,23].

A note on the status of the evidence discussed below is also required. This field combines mechanisms that are firmly established, such as the scaffolding role of PSD-95 and of the Shank family or the activity-dependent trafficking of AMPA receptors, with emerging evidence, such as the nanoscale alignment of trans-synaptic nanocolumns, and with working hypotheses that remain to be tested in human tissue, foremost among them the proposal that a pathological liquid-to-solid transition of synaptic condensates is a general driver of synaptic degeneration. We have attempted to signal these distinctions explicitly in the text, and hypotheses are introduced as such rather than as settled conclusions.

## 2. Molecular Architecture of the Post-Synaptic Density (PSD)

### 2.1. The Post-Synaptic Matrix: Homer-Shank-PSD-95 Interactomes as Structural Anchors

The ultrastructure of the excitatory postsynaptic density (PSD) represents a highly specialized, electron-dense thickening that anchors neurotransmitter receptors directly attached to the presynaptic active zone [27]. This architecture relies fundamentally on a polymeric network orchestrated by the scaffold proteins Homer and Shank [28]. Crystallographic analyses reveal that Homer-1b forms an antiparallel tetramer via intercalated, tail-to-tail dimeric coiled-coils. This unique tetrameric configuration, presenting dual EVH1 domains at both extremities, enables Homer to effectively crosslink multiple multimeric Shank hubs [29]. This coordinated assembly forms the PSD matrix: a flexible, high-order matrix, forming a highly scalable, flexible mesh-like network of structural and signaling proteins. This PSD matrix’s intricate, dynamic organization is essential for synaptic plasticity, regulating morphology, functional strength, and long-term stability [29,30,31]. Its mesh-like structure facilitates efficient diffusion and precise organization of receptors and ion channels, directly influencing the postsynaptic response to neurotransmitter release and enabling critical processes like learning and memory [32].

The scaffolding matrix structurally integrates with PSD-95 (DLG4), the most abundant membrane-associated guanylate kinase (MAGUK) in the PSD [33]. Bridging molecules, such as GKAP, stoichiometrically interlock the Homer–Shank matrix with PSD-95. Together, these structural proteins rigidly govern dendritic spine morphogenesis; experimental disruption of Homer tetramerization precipitates a collapse of Shank and PSD-95 synaptic localization, ultimately driving spine instability and severe synaptic depression [29].

### 2.2. Novel Hubs: The Significance of Interactors Like p140Cap and Rabphilin-3A in PSD Remodeling

Recent interactome mining has significantly expanded the known PSD protein network, revealing critical nodes intimately linked to the etiology of psychiatric synaptopathies. The adaptor protein p140Cap (encoded by the SRCIN1 gene) acts as a central hub for postsynaptic spine maturation and actin cytoskeleton remodeling [34]. At the PSD, p140Cap physically interacts with PSD-95, SNAP-25, and Src kinase, crucially inhibiting excitotoxic Src activation via the C-terminal Src kinase (Csk) [35]. The genetic ablation of p140Cap abolishes structural proteostasis, causing a failure in mature spine formation and promoting immature, filopodia-like protrusions, a morphological hallmark strongly associated with neurodevelopmental disorders [36].

Similarly, Rabphilin-3A (Rph3A), traditionally characterized solely as a presynaptic vesicle-associated Rab3A effector, exerts essential postsynaptic scaffolding functions [37]. Within the PSD, Rph3A interacts directly with the GluN2A subunit of the NMDA receptor and PSD-95 to form a trimeric stabilization complex. This specific structural alignment is heavily dependent on the C-terminal HVSSD motif of Rph3A binding directly to the PDZ3 domain of PSD-95 [37,38]. The complex formation acts as a localized coincidence detector, being heavily upregulated by the simultaneous presence of calcium ions and inositol triphosphate (IP3). In cortical and hippocampal circuits, Rph3A strictly partitions into mature, large-volume mushroom spines [39]. Pharmacological or genetic uncoupling of the Rph3A-GluN2A-PSD-95 ternary complex triggers rapid NMDAR endocytosis, spine shrinkage, and a complete blockade of long-term potentiation (LTP) [39].

### 2.3. Subsynaptic Compartmentalization: Nanoclusters and SSDs Observed via Super-Resolution

Advances in single-molecule localization microscopy (SMLM) and stimulated emission depletion (STED) have dismantled the traditional view of the PSD as a homogenous lattice, revealing a highly organized topology of subsynaptic domains (SSDs) [40,41]. SSDs are dynamic sub-compartments, ranging from 50 to over 130 nm in diameter, defined by densely packed local concentrations of specific scaffolding molecules and ionotropic receptors. PSD-95, alongside AMPA receptors, aggregates into these discrete SSDs, which undergo rapid morphological reorganization on a timescale of five to ten minutes in response to synaptic activity [42]. While smaller synapses generally maintain a single SSD, large, mature spines harbor multiple SSDs, correlating robustly with heightened AMPA receptor expression and enhanced synaptic strength [43].

**Figure 1:**
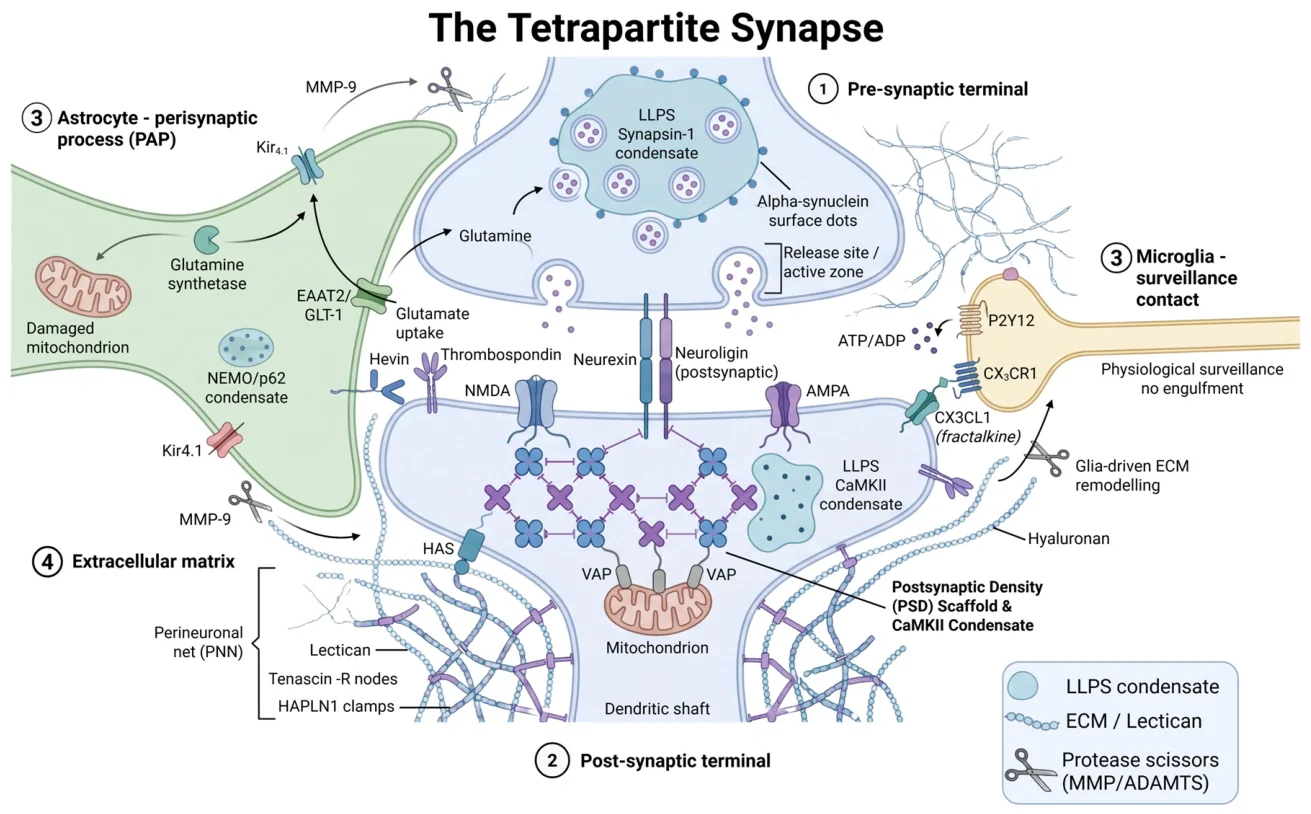
Structural organization of the tetrapartite synapse. Schematic representation of the four components of the tetrapartite synapse: (1) presynaptic terminal, containing a Synapsin-1 liquid–liquid phase separation (LLPS) condensate associated with the reserve vesicle pool; (2) postsynaptic compartment, comprising the postsynaptic density (PSD) scaffold (PSD-95, Homer, and Shank), a CaMKII LLPS condensate, glutamate receptors, and a VAP-tethered mitochondrion; (3) glial processes, including the perisynaptic astrocytic process (PAP), which mediates glutamate uptake and recycling, and a surveilling microglial process expressing P2Y12R, CX3CR1; and (4) the extracellular matrix (ECM), consisting of the perisynaptic ECM and the perineuronal net (PNN), organized by hyaluronan, lecticans, HAPLN1, and Tenascin-R and remodelled by MMP-9 and ADAMTS-4/5. Together, these neuronal, glial, and extracellular elements coordinate synaptic structure, signaling, and homeostasis.

Furthermore, 3D super-resolution mapping demonstrates that postsynaptic SSDs are structurally aligned with presynaptic active zone clusters, such as RIM1/2, to form “trans-synaptic nanocolumns” [40,41]. This transcellular nanoalignment is coordinated by trans-synaptic adhesion molecules like Neurexin and Neuroligin, optimizing the spatial proximity of vesicular fusion events to postsynaptic receptor nanoclusters [44]. The molecular compartmentalization into SSDs is further driven by the liquid–liquid phase separation (LLPS) of critical synaptic signaling enzymes like CaMKII [45]. Ultimately, this nanoscale compartmentalization establishes the fundamental functional unit of transmission, dynamically regulating structural plasticity and homeostatic resilience under both physiological and pathological conditions. Importantly, LLPS is not confined to the neuronal compartments: condensates form in the presynaptic terminal, in the postsynaptic density and in glial cells, and are therefore a property of all the cellular elements of the tetrapartite synapse (Figure 1).

## 3. Maintenance of Diversity: Proteostasis and Local Synthesis

### 3.1. Synaptic Local Translation: How mRNA Synthesis in Compartments Maintains Identity

Neurons are highly polarized cells that must asymmetrically distribute their proteome to establish distinct structural and functional domains, utilizing the active transport and local translation of mRNAs directly at target sites [46]. This local protein synthesis is not a marginal process. Recent high-throughput estimates indicate that approximately 50% of the local neuronal proteome is generated through the translation of localized transcripts, underscoring its dominance in maintaining synaptic homeostasis [47]. To achieve this extreme spatial compartmentalization, mRNAs are packaged into translationally repressed ribonucleoprotein (RNP) granules by RNA-binding proteins (RBPs) and transported via microtubule and actin cytoskeletal networks [48]. Upon local stimulation, such as NMDA receptor activation or depolarization, these dense RNP granules dynamically reorganize and dissolve, releasing the mRNA to be captured by the local translational machinery [49]. Advancing beyond the classical view that polyribosomes are exclusively required for active translation, some evidence suggests that a significant fraction of the synaptic translatome is managed by monosomes, providing a more efficient mechanism for localized synthesis [50].

This localized synthesis produces crucial plasticity proteins directly within dendritic spines and shafts, including CaMKII, PSD-95, AMPA receptor subunits (GluA1 and GluA2), and endogenous GABA_A receptor subunits. Furthermore, local translation establishes and maintains synapse-type-specific identities across distinct neural circuits [51,52]. For example, single thalamocortical projection neurons bifurcate to form distinct excitatory and inhibitory synapses, selectively routing and locally translating synaptic components based entirely on the specific identity of the postsynaptic target cell [53,54]. At the presynaptic terminal, localized mRNA translation is equally vital, supplying proteins such as SNAP25, RhoA, and Par3 to regulate synaptic vesicle dynamics, growth cone behavior, and active zone assembly [55]. This exquisite spatiotemporal autonomy is finely regulated by microRNAs (miRNAs) and the RNA-induced silencing complex (RISC), which undergo activity-dependent maturation locally at synapses to rapidly modulate the synthesis of target proteins on demand [55,56].

### 3.2. Liquid–Liquid Phase Separation: Biophysical Principles of Synaptic Condensates

Alongside localized translation, the spatial compartmentalization of pre- and postsynaptic signaling molecules is fundamentally governed by liquid–liquid phase separation (LLPS) [57]. LLPS enables the spontaneous segregation of biomolecules into coexisting condensed and dilute phases, forming dynamic, membrane-less microdomains that locally concentrate signaling components [58]. This biophysical process is driven by multivalent interactions among proteins containing intrinsically disordered regions (IDRs) or modular binding domains, such as SH3 domains [59]. At the pre-synapse, Synapsin-1 undergoes LLPS to cluster synaptic vesicles into a dynamic liquid reserve pool [60]. Simultaneously, active zone scaffolding proteins such as RIM and RIM-BP undergo LLPS to form dense condensates that recruit voltage-gated calcium channels (e.g., CACNA1) and tether synaptic vesicles precisely at the release site [59]. Furthermore, α-synuclein is dynamically recruited into these Synapsin-1 liquid droplets, where it remains in a liquid state to help organize the synaptic vesicle pool and regulate mobility [61,62].

In the postsynaptic density (PSD), LLPS similarly coordinates structural plasticity and the precise segregation of neurotransmitter receptors. NMDAR-mediated calcium influx triggers the phase separation of CaMKII with the GluN2B subunit and the primary scaffold protein PSD-95 [63]. This distinct CaMKII-GluN2B-PSD-95 condensate acts as a localized “sponge” that segregates AMPA and NMDA receptors into distinct nanodomains, coupling local calcium signaling to structural receptor retention [63]. Additionally, alternative splicing of G-protein coupled receptors adds another layer of regulation to this condensation; specific splice variants of latrophilin-3 containing PDZ-binding motifs drive the phase separation and robust clustering of critical postsynaptic scaffolds, including SHANK, PSD-95, GKAP, and HOMER [64,65]. The reversible nature of these condensates allows the PSD and active zone to rapidly assemble, reorganize, or dissolve in response to synaptic activity, providing a highly flexible architecture for synaptic transmission.

Condensate formation is not restricted to neurons. In astrocytes, oxidative and mitochondrial stress recruits the NF-κB essential modulator NEMO to damaged mitochondria, where it partitions into phase-separated condensates together with p62/SQSTM1 and nucleates the assembly of catalytically active IKK complexes, thereby coupling organelle damage to the transcription of inflammatory cytokines [66]. Glial regulation of the perisynaptic matrix is in turn sensitive to the biophysical state of its secreted and membrane-associated components, so that alterations of condensate dynamics in astrocytes and microglia may propagate to the synaptic environment described in Section 1.3 [22,25]. This glial dimension of LLPS is at present considerably less well characterized than its neuronal counterpart, and the causal link between glial condensate dysfunction and synapse loss should be regarded as a hypothesis under active investigation rather than as an established mechanism.

### 3.3. Mitochondrial Biogenesis: Local Energy Support for Synaptic Resilience

The compartmentalization and continuous remodeling of synapses during plasticity, including actin cytoskeleton turnover, the operation of ion pumps, and localized mRNA translation, demand massive amounts of ATP [67]. To meet these intense energetic demands, mitochondria are actively recruited and structurally anchored near dendritic spines and presynaptic boutons. The vesicle-associated membrane protein-associated protein (VAP) physically interacts with the actin cytoskeleton to stabilize mitochondria within specific dendritic microdomains of approximately 30 µm in length, creating a stable local energy grid [68]. These spatially stable dendritic mitochondria are strictly required to fuel local translation; experimental depletion of mitochondria from dendritic segments directly abolishes local protein synthesis and prevents the morphological enlargement of dendritic spines during long-term potentiation [49,69].

To maintain these local energy grids and sustain synaptic resilience, synaptic mitochondria rely heavily on decentralized local protein biogenesis [67]. High-resolution proteomic profiling and RNA sequencing of isolated synaptoneurosomes demonstrate that nuclear-encoded mitochondrial transcripts are transported to synapses and represent a massive fraction (approximately 24%) of all locally synthesized proteins following NMDA receptor stimulation [67]. Crucially, these newly synthesized mitochondrial proteins are not merely bystanders; they are actively imported into synaptic mitochondria and incorporated into functional respiratory chain supercomplexes, including Complex I, II, III, IV, and ATP synthase [67]. The clinical relevance of this local biogenic pathway is starkly highlighted by neurodevelopmental synaptopathies. In Fmr1 knockout mice, a model for Fragile X Syndrome characterized by the genetic dysregulation of local synaptic translation, profound abnormalities emerge in synaptic mitochondrial morphology [67,70]. These dysregulated synapses exhibit small, round mitochondria with altered cristae arrangements, dense matrices, and aberrant respiration rates, also due to the impaired oxidative phosphorylation and respiratory defects, demonstrating that the local translation of mitochondrial constituents is absolutely essential for maintaining the bioenergetic resilience of the synapse [67,71].

Decoding how diverse synaptic proteoforms are distributed across the brain requires a transition from bulk tissue studies to high-dimensional, single-synapse technologies, such as mass synaptometry and spatial omics.

### 3.4. Mapping Technologies to Identify Vulnerable Neuronal Populations in Neurodegenerative Diseases

To decode the extreme molecular heterogeneity of individual synapses, researchers have adapted cytometry by time-of-flight mass spectrometry (CyTOF) to analyze enriched nerve terminals, a technique termed Mass Synaptometry [72]. This adaptation enables the simultaneous, highly parallel molecular profiling of up to 34 distinct parameters across tens of thousands of individual synapses in a single assay [72,73]. The application of Mass Synaptometry to human post-mortem striatal samples successfully identified highly restricted dopaminergic transporter (DAT) expression within specific presynaptic clusters, highlighting broad disease-specific derangements in the molecular composition of synaptic terminals in Lewy Body Disease [74,75].

The limitations of classical affinity purification in capturing transient, low-affinity protein–protein interaction (PPIs) within intact circuits have been circumvented by the advent of spatially restricted, proximity-dependent biotinylation. BioID and its faster derivative TurboID covalently biotinylate proteins within a radius of 10 to 20 nanometers of a targeted bait [76,77,78,79,80]. To capture dynamic intercellular communications, such as the glia-neuron cross-talk within the tetrapartite synapse, the Split-TurboID approach was engineered [81,82]. By applying Split-TurboID in vivo, researchers successfully isolated the astrocyte–neuron interface interactome, revealing that the neuronal cell adhesion molecule NRCAM strongly localizes to perisynaptic astrocytic processes to govern inhibitory synapse organization [83].

Isolating pure, cell-type-specific synaptic fractions from highly intermingled brain tissue requires advanced flow cytometric techniques, most notably Fluorescence-Activated Synaptosome Sorting (FASS) [12,84]. FASS purifies intact synaptosomes encompassing the presynaptic active zone, synaptic vesicles, and the attached postsynaptic density, enabling deep proteomic identification of previously uncharacterized synapse-specific components like FXYD6 and Tpd52 [81,84]. Furthermore, applying FASS to dopaminergic terminals revealed the existence of specialized “dopamine hub synapses” that contain multiple synaptic compartments, fundamentally altering our understanding of volume transmission [85]. To anchor this synaptic diversity within the anatomical architecture of the brain, spatial transcriptomics (ST) is increasingly integrated with single-nucleus RNA sequencing (snRNA-seq). This spatial omics approach identifies vulnerable neuronal populations in neurodegenerative states, demonstrating that ST spots corresponding to specific cortical niches exhibit dynamic shifts in gene expression intimately linked to amyloid pathology accumulation [86,87,88]. Layer- and cell-type–specific (LCS/CLS-CTS) differential expression analyses reveal AD-associated genes (e.g., APOE, KCNIP3, and CTSD) that remain undetectable when using non-spatial snRNA-seq alone [88]. Consistently, spatial transcriptomics (ST) spots enriched for amyloid plaques display coordinated plaque-induced gene (PIG) networks, oligodendrocyte (OLIG) responses, and glial activation modules, whose intensity and composition vary across cortical layers and anatomical regions [87,89,90,91].

Within this framework, spatially stratified “plaque–glia niches” identified in the human AD cortex delineate distinct glial states and patterns of neuronal loss between low-Aβ and glia-high areas, thereby tightly linking local transcriptional remodeling to Aβ burden and synaptic degeneration [91,92,93]. In line with these observations, multi-region ST analyses further demonstrate a region-specific vulnerability of inhibitory neuronal subtypes, together with alterations in endo-lysosomal and metal-homeostasis genes occurring within Aβ-rich microenvironments [89,94,95].

By leveraging these advanced mapping technologies, researchers have begun to unravel the ’molecular logic’ behind selective neuronal vulnerability. The ability to isolate specific synaptic populations via FASS and profile their unique interactomes has revealed that neurodegeneration does not progress through a uniform collapse. Instead, it is a targeted process where pathological proteins exploit the baseline molecular signatures of high-risk synaptic niches, such as the RORB+ excitatory clusters, as observed in the earliest stages of Alzheimer’s Disease.

## 4. Selective Vulnerability in Alzheimer’s Disease

### 4.1. Oligomer Interactomes: How Aβ and Tau Target Specific Protein Complexes

Before turning to specific mechanisms, it is useful to state why the postsynaptic density and soluble oligomeric species are given priority in this and the following section, as this choice shapes what follows. Three considerations motivate it.

First, among the structural correlates of cognition, the loss of postsynaptic scaffolds and of dendritic spines tracks clinical impairment more closely than classical aggregate measures in Alzheimer’s disease [96]. Lower spinophilin-positive spine density and lower spinophilin levels are associated with both worse cognition and greater Aβ plaque burden, supporting the idea that synaptic structure is closer to clinical status than plaque burden alone [97]. Dendritic spine density in dorsolateral prefrontal cortex is reduced in symptomatic AD, while spine density is not associated with neuritic plaque score and instead shows a negative correlation with Braak stage [98]. Across the AD continuum, in vivo synaptic density is a significant predictor of performance across multiple cognitive domains [99].

Second, the PSD is the compartment in which the diseases converge morphologically because it is a scaffold-rich protein network that organizes excitatory synapses and is disrupted across neurodegenerative dementias [99,100]. Within that network, PSD-95/MAGUK–GKAP–Shank interactions define the core scaffold [99,100]. In AD cortex, isolated PSDs show major alterations in glutamate receptors PSD-95, Shank3, and SynGAP. Gong and Lippa explicitly argue that PSD destruction may be a common mechanism in selectively vulnerable regions across AD, DLB, and FTD [100]. Tau also directly perturbs PSD-like assemblies by arresting condensates formed by PSD-95, GKAP, Shank, and Homer, linking disease protein accumulation to the same scaffold hub [101].

Third, soluble oligomers rather than mature fibrils are a tractable mechanistic entry point because acute toxicity is often assigned to diffusible species that directly perturb neuronal membranes and synapses [96]. For α-synuclein, fibrils can act as reservoirs that release soluble prefibrillar oligomers, and the kinetics of oligomer release match the kinetics of toxicity in neurons [102]. Reviews of synucleinopathy evidence therefore emphasize that soluble oligomeric species are often more toxic than larger fibrillar forms, even though aggregate-state toxicity remains heterogeneous and context-dependent [102].

This focus is deliberate and necessarily partial: presynaptic, axonal, and myelin-related contributions to selective vulnerability are also well documented [103]. AD vulnerability networks are enriched for processes associated with axonal remodeling [104], and recent human and mouse proteomics identifies the myelin–axon interface as a site of amyloid-linked disrupted neuro-glial signaling [103].

In the early stages of Alzheimer’s disease, synaptopathy does not occur uniformly; rather, specific neuronal populations exhibit profound selective vulnerability driven by intracellular protein accumulation [105]. High-dimensional spatial transcriptomics reveals that layer 5 and 6 RORB + FOXP 2+ excitatory neurons, alongside layer 3, 5, and 6 GAD1 + FOXP 2+ inhibitory neurons, exhibit immediate and severe degeneration directly corresponding to the early intracellular accumulation of Aβ (intraAβ) [106]. Conversely, other populations such as layer 3 RORB + GPC 5 + neurons act as primary hubs for early phosphorylated Tau (pTau) accumulation but display a paradoxical resilience to immediate cell death [106].

The transition from neuronal resilience to vulnerability is dictated by the pathological protein–protein interaction (PPI) networks, or interactomes, of soluble Aβ and Tau oligomers. At the excitatory postsynaptic density (PSD), current evidence indicates that Aβ oligomers can act as potent extracellular zinc chelators; the sequestration of cleft Zn^2+^ is proposed to prevent the zinc-dependent multimerization of the ProSAP2/Shank3 scaffolding matrix and thereby to destabilize the PSD architecture [107]. Zinc occupies a dual position at excitatory synapses. Vesicular Zn^2+^ is stored in a subset of glutamatergic terminals in the hippocampus and cortex, is co-released with glutamate during activity, and can transiently reach cleft levels from micromolar to well above 100 μM [108]. At the same time, synaptic zinc modulates NMDA receptors through a high-affinity site on GluN2A, and acute stimulation transiently inhibits postsynaptic GluN2A-NMDAR signaling [108,109].

That makes zinc both a scaffold cofactor and a receptor modulator. Shank proteins form a core PSD-95/GKAP/Shank/Homer scaffold deep in the PSD, and zinc-dependent Shank assembly provides the structural side of this mechanism, while zinc-sensitive NMDAR gating provides the electrophysiological side [110]. Aβ binds Zn^2+^ in the low micromolar range, and zinc also alters Aβ aggregation and can promote distinct toxic oligomeric states, making zinc sequestration a plausible link between early scaffold destabilization and receptor dysfunction [111]. Most of that support comes from in vitro, ex vivo, modeling, or acute-slice systems, while human evidence more directly supports early Aβ-associated postsynaptic NMDAR/PSD changes than the relative contribution of zinc sequestration itself [112].

Concurrently, Aβ oligomers bind directly to the lipid-anchored cellular prion protein (PrPc) at the synaptic membrane, which subsequently recruits and hyperactivates the tyrosine kinase Fyn [1,113]. Pathological Tau synergizes with this excitotoxic cascade by mislocalizing to the dendritic spine, where it functions as an aberrant scaffold that directly couples Fyn to the PSD-95/NMDAR complex, driving the hyperphosphorylation of the GluN2B subunit [114,115].

Presynaptically, the pathological Tau interactome directly targets the synaptic vesicle (SV) cycle. The N-terminal domain of hyperphosphorylated Tau binds abnormally to the vesicular protein Synaptogyrin-3 (Syngr3), forming a pathological complex that restricts SV mobility and rapidly exhausts neurotransmitter release during sustained depolarization [116]. Furthermore, specific aberrant post-translational modifications of Tau, notably acetylation at K281 and K274, precipitate a severe deficiency of the postsynaptic Kidney/BRAin (KIBRA) protein. This loss abolishes activity-dependent F-actin polymerization, physically uncoupling AMPA receptors from the cytoskeletal matrix [117,118,119].

### 4.2. Receptor Dysregulation: Trafficking Failure of AMPA and NMDA Receptors

The structural disruption produced by the Aβ and Tau interactomes manifests physiologically as a progressive failure of glutamate receptor trafficking. Under healthy conditions, the retention of AMPA receptors within subsynaptic domains (SSDs) relies on a mechanism known as diffusional trapping, where activated CaMKII phosphorylates the transmembrane AMPA receptor regulatory protein (TARP) stargazin to tightly bind the PSD-95 scaffold [120]. However, prolonged exposure to Aβ oligomers overactivates extrasynaptic GluN2B-containing NMDARs, which dominantly sequester active CaMKII and prevent its activity-dependent translocation to the synaptic cleft [121,122].

This deprivation of synaptic CaMKII shifts the local biochemical balance heavily toward phosphatase activity, reversing the phosphorylation of stargazin and untethering the AMPA receptor complex from PSD-95 [123]. The subsequent lateral diffusion and endocytosis of AMPA receptors leads to early, profound long-term depression (LTD) and synaptic silencing [120]. Receptor trafficking is further compromised by the promiscuous binding of Aβ oligomers to an array of modulatory surface receptors, including mGluR5 and EphB2, which chronically disrupts intracellular calcium homeostasis and completely abolishes the induction of long-term potentiation [124,125].

### 4.3. Complement-Mediated Synaptopathy: The Role of C1q and Glia-Neuron Cross-Talk

The two preceding subsections focused on cell-autonomous events. However, as outlined in Section 1.3, the synapse is a tetrapartite structure whose stability also depends on glial cells and the extracellular environment. These mechanisms therefore represent the stage at which Aβ- and Tau-induced vulnerability progresses to irreversible structural loss.

The failure of localized proteostasis and receptor anchoring triggers a non-cell-autonomous clearance mechanism governed by the tetrapartite synapse. As vulnerable neurons accumulate pathological oligomers, their synapses undergo severe metabolic stress; terminals exhibiting high levels of mitochondrial reactive oxygen species are selectively tagged by the classical complement initiation factor C1q [126]. The vulnerability to complement tagging is further exacerbated by structural abnormalities within the presynapse, as an aberrant accumulation of Septin-3 and Septin-5 polymers strongly correlates with massive C1q deposition [26,126].

Once tagged by C1q, the classical complement cascade deposits C3 onto the synaptic membrane, creating a potent “eat-me” signal that triggers pathological pruning by surrounding glial cells [127]. High-resolution 3D reconstructions in tauopathy models reveal a striking molecular division of labor during this glia–neuron cross-talk: reactive astrocytes predominantly orchestrate the engulfment of excitatory Homer1-positive synapses in a strictly C1q-dependent manner [128]. Conversely, microglial lysosomes exhibit a distinct proclivity for phagocytosing inhibitory gephyrin-positive subsynaptic domains [128].

The trajectory of this complement-mediated synaptopathy is heavily influenced by AD genetic risk variants expressed in the glial compartment. In models harboring loss-of-function mutations in the microglial receptor TREM2, microglia are severely impaired in their ability to phagocytose damaged synapses surrounding Aβ plaques. In response to this microglial failure, astrocytes mount a maladaptive compensatory response, dramatically increasing their engulfment of inhibitory synapses [129,130]. This aberrant, glia-driven elimination of inhibitory input directly contributes to the characteristic network hyperexcitability and cognitive decline that defines early-stage Alzheimer’s disease [131].

**Figure 2:**
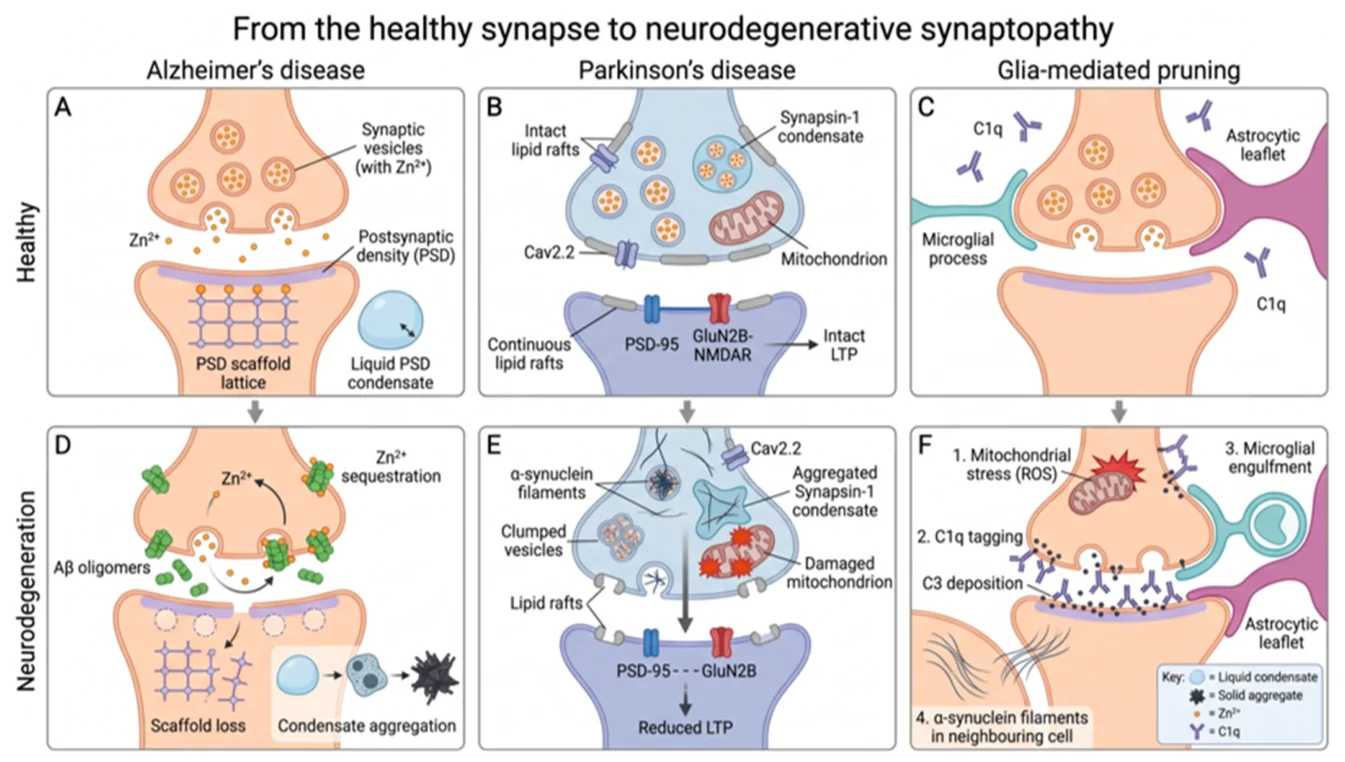
From the healthy synapse to neurodegenerative synaptopathy. Each column compares a healthy synapse (top row) with its degenerating counterpart (bottom row). (**A**) At the healthy excitatory synapse, Zn^2+^ stored in synaptic vesicles is released into the cleft and occupies the postsynaptic scaffold lattice, whose Zn^2+^-dependent assembly maintains the architecture of the postsynaptic density; the associated PSD condensate is a dynamic liquid phase in free exchange with the surrounding cytosol. (**D**) In Alzheimer’s disease, soluble Aβ oligomers act as extracellular zinc chelators and sequester cleft Zn^2+^, leaving the metal-binding sites unoccupied (dashed outlines) and causing loss of the scaffold lattice. In parallel, the PSD condensate aggregates, passing from a dynamic droplet through a gel-like intermediate to an insoluble solid aggregate. (**B**) At the healthy presynaptic terminal, intact and continuous lipid raft microdomains retain the Cav2.2 channel, synaptic vesicles are held in a liquid Synapsin-1 condensate and mitochondria are intact; postsynaptically, PSD-95 remains coupled to the GluN2B-containing NMDA receptor within rafts, preserving long-term potentiation. (**E**) In Parkinson’s disease, α-synuclein filaments fragment the presynaptic rafts and displace Cav2.2, partition into the Synapsin-1 condensate and drive its aggregation, and are accompanied by vesicle clumping and mitochondrial damage. Raft fragmentation propagates across the cleft: postsynaptic rafts break up in turn, PSD-95 and GluN2B are uncoupled and long-term potentiation is reduced. (**C**) Under physiological conditions, microglial processes and astrocytic leaflets survey the intact synapse without depositing complement, and C1q remains unbound in the extracellular space. (**F**) When the synapse becomes stressed, mitochondrial reactive oxygen species (1) precede C1q tagging of the synaptic membrane (2) and C3 deposition as an “eat-me” signal (3), culminating in microglial engulfment of part of the synapse (4); α-synuclein filaments in a neighboring cell act as an upstream trigger.

While the primary protein aggregates differ between Alzheimer’s and Parkinson’s diseases, a convergence emerges at the level of the postsynaptic density (PSD) [105], and it is this convergence, rather than any shared etiology, that the title of this review refers to. Both Aβ/Tau interactomes and pathological α-synuclein appear to exert part of their toxicity by destabilizing the same scaffolding hubs—specifically PSD-95 and the Shank family—that maintain synaptic identity. Whether through zinc sequestration in AD or lipid raft fragmentation in PD, the resulting synaptic disruption appears to follow a comparable trajectory, namely the uncoupling of ionotropic receptors from their structural anchors, which is associated with progressive network dysfunction well before overt neuronal loss (Figure 2). Stated precisely, the convergence proposed here is one of downstream molecular target and of biophysical mechanism, not of upstream cause: the two diseases begin differently and end, at the level of the PSD, in a comparable state.

## 5. Synaptic Dysfunction in Parkinson’s Disease

### 5.1. A-Synuclein Toxicity: Pre- and Post-Synaptic Disruption via Lipid Raft Fragmentation or Interactome Interference

Normally, α-Synuclein (α-Syn) binds to curved phospholipidic membranes and partitions specifically into cholesterol-rich lipid rafts [132]. However, pathological exposure to exogenous monomeric α-Syn physically disrupts these dynamic microdomains, fragmenting lipid rafts into dysfunctional, isolated structures ranging from 0 to 5 × 10^−3^ µm^2^ [133]. This mechanical fragmentation structurally displaces critical synaptic proteins, such as presynaptic Cav2.2 calcium channels, into cholesterol-poor regions, triggering aberrant calcium influx and acute synaptic vesicle (SV) mobilization [134]. Simultaneously, postsynaptic stability is compromised as the major scaffold PSD-95 and the NMDA receptor subunit NR2b (GluN2B) are displaced from lipid rafts, with disruption of the NMDAR-PSD-95 interaction and a consequent decrease in long-term potentiation [133].

Beyond direct membrane disruption, α-Syn toxicity is driven by pervasive interactome interference that sabotages SV proteostasis and cycling. Under physiological conditions, the presynaptic SV pool is organized into a functional liquid condensate orchestrated primarily by the liquid–liquid phase separation (LLPS) of Synapsin-1 [135]. Native α-Syn is recruited into these Synapsin-organized liquid droplets, remaining in a fluid state to regulate SV clustering and mobility. Under pathological conditions, however, abnormally high concentrations of α-Syn are thought to undergo an aberrant liquid-to-solid phase transition [136]. Direct biophysical support for this step is now available: α-Syn aggregation nucleates through LLPS, and droplets generated both in vitro and in cells mature over time into an amyloid hydrogel containing oligomeric and fibrillar species, with the factors that aggravate aggregation in disease, including low pH, phosphomimetic substitution and familial mutations, also accelerating droplet formation and maturation [136,137]. This phase transition physically hardens the condensate, sequestering SNARE components like VAMP2 and effectively arresting SV exocytosis [138].

### 5.2. Hippocampal vs. Striatal Failure: Early Synaptic Failure as a Driver of Cognitive Deficits

While PD is classically defined by striatal dopaminergic loss, early synaptic failure within the hippocampus operates as a primary driver of the subtle cognitive deficits that often emerge during the prodromal phase of the disease [139]. In viral vector models, A53T-mutated human α-Syn spreads rapidly from the ventral tegmental area (VTA) to dopaminergic, glutamatergic, and GABAergic axon terminals in the hippocampus [139]. This pathological infiltration precipitates a profound impairment in hippocampal long-term potentiation (LTP) by the first week post-inoculation, significantly preceding actual cognitive decline. This early failure of hippocampal LTP is driven by localized proteostatic dysregulation, marked specifically by an enhanced basal expression of the GluA1 AMPAR subunit at the postsynaptic membrane [139].

Conversely, within the striatum, the progressive depletion of dopamine alters the physiological composition and synaptic enrichment of NMDARs on spiny projection neurons (SPNs) [140]. Partial dopaminergic denervation induces a selective increase in GluN2A synaptic levels, which directly impairs striatal LTP and drives the onset of early motor abnormalities [141].

### 5.3. PSD Reorganization: Redistribution of SAP97 and PSD-95 in PD Models

The collapse of synaptic plasticity in PD is inextricably linked to the structural reorganization of the postsynaptic density (PSD) and the spatial redistribution of major PSD-MAGUK scaffolding proteins [30]. In experimental animal models of PD, toxic interactomes induce the subcellular redistribution of PSD-95 and SAP97, dismantling the structural framework of postsynaptic excitatory domains [142]. In advanced denervation models, there is a significant reduction in the physiological association between PSD-95 and NMDARs, coupled with an aberrant increase in αCaMKII binding to both the GluN2A and GluN2B subunits [143]. The destabilization of PSD-95 interactions is exacerbated by the loss of protective binding from Rabphilin-3A (Rph3A), a critical postsynaptic hub protein that normally stabilizes the GluN2A/PSD-95 complex within mature subsynaptic domains [39]. Demonstrating the translational relevance of stabilizing these protein–protein interaction networks, the administration of cell-permeable peptides that structurally uncouple the aberrant GluN2A/PSD-MAGUK interaction effectively restores physiological synaptic composition and significantly reduces dyskinetic motor behaviors [141,144].

### 5.4. Genetic Modifiers, Glial Signaling and Phase Behavior in Parkinsonian Synaptopathy

The mechanisms described so far derive largely from models of α-synuclein excess. A complementary body of work indicates that the commonest genetic determinants of Parkinson’s disease converge on the same presynaptic machinery. Mutations in LRRK2 and GBA1, together with the alterations of the Rab family of small GTPases that both proteins regulate, disrupt synaptic vesicle trafficking, endolysosomal sorting and autophagy at the terminal, and this early synaptopathy is thought to drive retrograde, terminal-to-soma degeneration rather than to follow it [145]. Consistent with this interpretation, treatment of human-induced pluripotent stem cell-derived dopaminergic neurons carrying GBA1, LRRK2 or VPS35 mutations with N-acetyl-L-leucine reduced Ser129-phosphorylated α-synuclein in an HTRA1-dependent manner and increased wild-type parkin, with measurable improvement in synaptic vesicle endocytosis in vitro and in dopamine-dependent motor learning in LRRK2 R1441C knock-in mice [146]. Presynaptic dysfunction therefore appears to be an early and, in principle, modifiable node rather than a terminal event.

The glial component described for Alzheimer’s disease is operative here as well, although it has been characterized in less detail. LRRK2 is expressed predominantly in microglia, where it shapes innate immune responses; its genetic ablation in the 5xFAD model reduced C1qa and C3 expression at both excitatory and inhibitory synapses, preserved PSD-95 levels and prevented synaptic loss, indicating that a Parkinson’s disease risk gene can act on synapses through a complement-dependent glial mechanism shared by the two disorders [147]. This is among the more direct pieces of evidence for the convergence proposed in Section 4.3, and it suggests that the neuroimmune strategies discussed in Section 7.3 need not be restricted to amyloid-driven pathology.

Finally, the link between Parkinsonian synaptic failure and phase behavior is supported by a second, independent example. The parkin-interacting substrate PARIS (ZNF746) undergoes LLPS through its N-terminal low-complexity domain and is driven towards an amorphous solid state by poly(ADP-ribose) binding to its C-terminal prion-like domain; SDS-resistant, solidified PARIS species are detectable in the substantia nigra of mice injected with α-synuclein preformed fibrils and in adult conditional parkin knockouts, and are prevented by PARP1 deficiency [148]. Together with the α-synuclein data above, this supports, without yet establishing, the more general proposition advanced in this review that the transition from a liquid to a solid condensate state is a shared pathological step in the two diseases [136,137].

## 6. Vascular and Peripheral Contributions to Synaptic Vulnerability

The account given so far is, deliberately, a cell-biological one. It is increasingly clear, however, that both Alzheimer’s and Parkinson’s disease involve systemic alterations that act on the same synaptic structures from outside the neuropil, and a review organized around the tetrapartite synapse cannot ignore the vascular compartment that supplies it. The blood–brain barrier, formed by brain endothelial cells and supported by pericytes and astrocytic end-feet, deteriorates early in both disorders, and regional barrier breakdown in the hippocampus is detectable in cognitively normal individuals and predicts subsequent decline independently of amyloid and tau biomarkers [149,150]. Barrier failure permits the entry of plasma-derived proteins such as fibrinogen, thrombin and plasminogen into the perivascular space, where they activate microglia and promote degradation of the perisynaptic matrix described in Section 1.3, while pericyte loss reduces cerebral blood flow and impairs vascular clearance of Aβ [149,150]. In this sense, the vascular compartment is not a parallel pathology but an upstream determinant of the glial and matrix states that govern synaptic stability.

Peripheral contributions follow a similar logic. Platelets are the principal peripheral source of amyloid precursor protein and also contain α-synuclein, tau and the secretases ADAM10 and BACE1; their activation state and protein content are altered in dementia, which has motivated their evaluation as accessible peripheral biomarkers [151]. Whether platelet-derived Aβ contributes materially to the cerebral pool remains unresolved, but the co-occurrence of platelet activation, endothelial dysfunction and barrier leakage provides a plausible route by which a peripheral process could modify the synaptic environment. Peripheral immune signaling adds a further layer, since circulating complement components, cytokines and infiltrating lymphocytes can reach the perivascular compartment once barrier integrity is compromised, and thereby amplify the C1q- and C3-dependent mechanisms described in Section 4.3 and Section 5.4.

## 7. Precision Pharmacology and Targeted Synaptic Stabilization

### 7.1. Targeting Pathological Phase Transitions: Restoring Condensate Fluidity

If the loss of condensate fluidity is indeed a step common to the two diseases, then the phase behavior of synaptic proteins is in principle a therapeutic target, and this section therefore begins with it rather than treating it as an afterthought. The rationale is biophysical. Condensates are held together by weak, multivalent interactions between intrinsically disordered regions and modular domains, and these interactions are, unlike a folded binding pocket, tunable: post-translational modifications, molecular chaperones and small molecules that partition into the dense phase can shift the balance between the liquid and the solid state without abolishing the assembly itself. The therapeutic objective is therefore not to dissolve synaptic condensates but to preserve their material properties.

**Figure 3:**
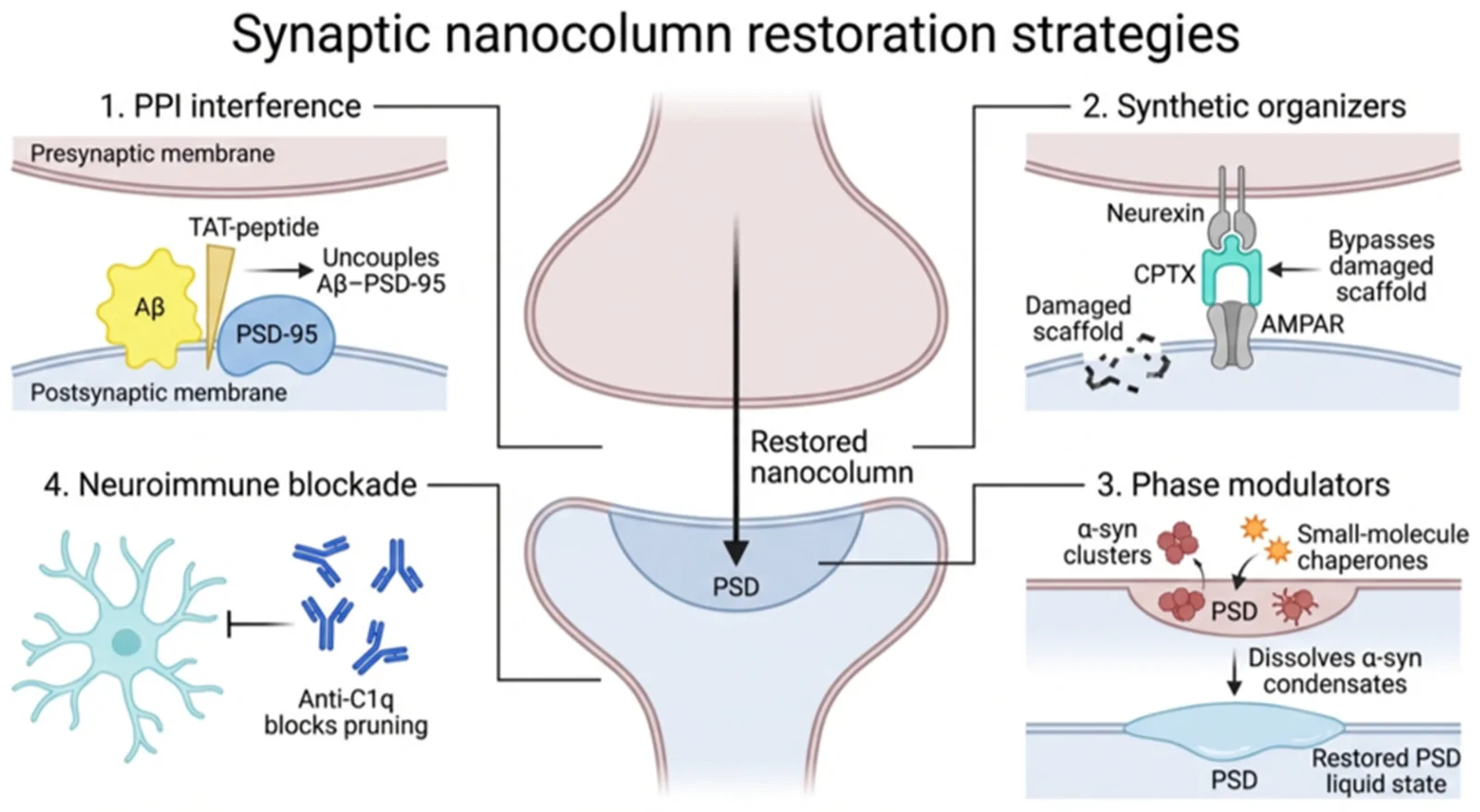
Therapeutic Frontiers in Precision Synaptopharmacology. (1) PPI Interference: Gold-coded cell-penetrating peptides (e.g., TAT-peptides) disrupt toxic protein–protein interaction (e.g., Aβ-scaffold). (2) Synthetic Organizers: Teal-colored CPTX acts as a molecular bridge to physically re-align the trans-synaptic nanocolumn. (3) Phase Modulators: Small-molecule chaperones (orange) reverse pathological protein hardening, restoring the physiological fluidity and LLPS dynamics. (4) Neuroimmune Blockade: Monoclonal antibodies (royal blue) shield synaptic terminals from C1q-mediated microglial engulfment, preserving circuit connectivity.

Three lines of work define the current state of this field. First, molecular chaperones of the HSP70 family and the small heat-shock proteins act on precisely the maturation step that converts a liquid droplet into a gel or a fibril, and their pharmacological induction is being explored as a means of maintaining condensate fluidity. Two examples make the principle concrete. A defined human disaggregase, formed by Hsc70 together with the class B J-protein DNAJB1 and an Hsp110 nucleotide-exchange factor, disassembles preformed α-synuclein amyloid fibrils within minutes in vitro by combined fragmentation and depolymerization, regenerating non-toxic monomers [152]. In parallel, the HSPB8-BAG3-HSP70 complex acts as a surveillance module that preserves the dynamism of phase-separated granules and prevents their conversion into immobile, aggregate-prone structures [153]. A conceptually related strategy exploits endogenous proteins that behave as phase-reversal chaperones: nuclear-import receptors such as Karyopherin-beta2 both prevent and reverse the fibrillization of prion-like-domain proteins and dissolve aberrant hydrogels, restoring the liquid state [154]. The same logic underlies the phase-stabilizing chaperones represented in Figure 3. Second, systematic screening platforms are beginning to make condensates tractable as a target class: an integrated high-content and genome-wide CRISPR screening pipeline recently identified small molecules that prevent the pathological accumulation of a reporter protein into nuclear condensates in a cellular model of DYT1 dystonia, and correlated condensate abundance with genes implicated in several neurodevelopmental disorders [155]. Third, the same biophysical logic applies to the aggregating species themselves, since compounds that partition into α-synuclein droplets could in principle delay the liquid-to-solid transition described in Section 5.4. The oligomer modulator anle138b illustrates how far this approach has advanced: identified by high-throughput screening and optimized medicinally, it binds pathological aggregates in a structure-dependent manner, blocks oligomer formation by α-synuclein and prion protein both in vitro and in vivo, and reduces oligomer accumulation, neuronal degeneration and disease progression in three Parkinson’s disease mouse models, with good oral bioavailability and blood–brain barrier penetration [156]. It should be noted that anle138b targets the aggregation pathway rather than the material state of a physiological synaptic condensate, so it exemplifies the pharmacological feasibility of the approach rather than its direct application to the postsynaptic scaffold.

These possibilities should be stated with the appropriate reservation. No condensate-directed agent has yet entered clinical development for a synaptopathy, and the selectivity of such compounds is at present poorly defined: the same weak, multivalent chemistry that makes condensates tunable also makes them difficult to address selectively, since a molecule that alters the material state of one condensate may well alter others in the same cell. Targeting phase behavior is, for the moment, a well-motivated hypothesis supported by encouraging preclinical tools, and not an established therapeutic strategy. The interventions described in the following subsections are at present more advanced, and several of them act, indirectly, on the same scaffolding equilibria.

### 7.2. Precision Synaptopharmacology: Targeted Disruption of Pathological Protein–Protein Interactions

The structural architecture of the postsynaptic density relies heavily on the specific binding of NMDAR regulatory subunits (GluN2A and GluN2B) to PSD-MAGUK scaffolding proteins via their C-terminal domains [157,158]. In neurodegenerative states such as Parkinson’s disease and L-DOPA-induced dyskinesia, aberrant stabilization of the GluN2A/PSD-95 complex drives pathological NMDAR synaptic enrichment and motor abnormalities [38,144,158]. In this context of therapy, the administration of cell-permeable peptides designed to physically uncouple GluN2A from PSD-MAGUKs effectively restores physiological NMDAR composition and significantly reduces dyskinetic behaviors [38,159]. Indeed, disrupting the GluN2A/PSD-95/Rph3A complex with TAT-based peptides used as “Tat-based therapies” has been investigated as new a pharmacological alternative to treat these pathologies. Tat is an 11–amino acid, arginine/lysine-rich sequence from HIV-1 (residues 47–57) that functions as a cell-penetrating peptide (CPP), able to cross cell membranes and even the blood–brain barrier (BBB) and drag attached “cargo” molecules with it [160,161,162]. Consistently, the interfering peptide TAT-2A-40, which selectively uncouples GluN2A from Rph3A (and the broader GluN2A/PSD-95/Rph3A scaffold), decreases synaptic GluN2A availability and lowers the GluN2A/GluN2B ratio, resulting in impaired long-term potentiation (LTP) and spatial memory [38,159]. In contrast, Tat-NR2B9c and AVLX-144, by disrupting the GluN2B–PSD-95–nNOS complex, attenuate L-DOPA-induced dyskinesia and confer neuroprotection in stroke while preserving physiological NMDAR signaling [163].

In the context of acute ischemic synaptopathy, precision targeting of the GluN2B interactome is achieved via the NA-1 peptide (nerinetide), a sequence comprising the nine C-terminal residues of the GluN2B subunit [164,165]. NA-1 competitively disrupts the physical association between GluN2B, PSD-95, and neuronal nitric oxide synthase (nNOS), thereby attenuating neurotoxic calcium signaling without blocking the physiological pro-survival ion flux of the NMDAR [163,166]. Conversely, in psychiatric synaptopathies characterized by NMDAR hypofunction, the cell-penetrating TAT-SAPIP peptide is utilized to target the Src/PSD-95 interaction. By preventing PSD-95 from binding to the Src SH2 domain, TAT-SAPIP disinhibits Src kinase activity, subsequently enhancing synaptic NMDAR currents and rescuing cognitive deficits in trace fear conditioning models [167].

### 7.3. Neuroimmune Targets: Anti-C1q Antibodies and Inhibition of Microglial Synaptic Stripping

Because complement-dependent pruning was identified in Section 1.3, Section 4.3 and Section 5.4 as the final common effector through which a destabilized synapse is physically removed, blocking that step is a logical counterpart to the scaffold- and phase-directed strategies described above rather than a separate therapeutic theme.

The aberrant reactivation of the classical complement cascade mediates early synapse loss across multiple neurodegenerative models, positioning the initiator protein C1q as a primary target to halt pathological synaptic pruning [26]. Pharmacological intervention using the anti-C1q monoclonal antibody ANX-M1 effectively blocks the activation of this cascade in the presence of synaptotoxic stressors [168]. In wild-type mice exposed to soluble Aβ oligomers, pre-treatment with ANX-M1 prevented microglial synaptic engulfment, preserved synaptic density, and completely abolished the oligomer-induced impairment of long-term potentiation (LTP) [26]. The translational potential of this neuroimmune modulation is currently being evaluated in clinical settings, with humanized variants like the systemic ANX005 and the ophthalmic ANX-007 entering clinical trials [169]. A Phase 2 open-label trial utilizing ANX005 in patients with Huntington’s disease successfully suppressed C1q levels in the cerebrospinal fluid, demonstrating robust target engagement within the central nervous system [170,171]. At the preclinical level, preventing the downstream deposition of the C3 opsonin mimics this neuroprotection. Genetic ablation of either C1q or C3 in the APP/PS1 and TauP301S models radically reduces the volume of excitatory (Homer1) and inhibitory (gephyrin) synaptic puncta internalized by both microglial and astrocytic lysosomes, preserving overall synaptic density despite the presence of advanced proteinopathy [128].

### 7.4. Synthetic Synaptic Organizers: Molecular “Bridges” to Restore Glutamatergic Circuits

To actively reconstruct disrupted synaptic architectures, researchers have engineered synthetic extracellular scaffolding proteins (ESPs) using structure-guided design principles [172]. The synthetic synaptic organizer CPTX was constructed by fusing the presynaptic Neurexin-binding cysteine-rich region (CRR) of Cerebellin-1 (Cbln1) with the postsynaptic AMPA receptor-binding pentraxin (PTX) domain of Neuronal Pentraxin-1 (NP1) [173]. CPTX maintains a stable hexameric conformation and acts as a bidirectional trans-synaptic bridge, strictly interacting with the presynaptic Neurexin-1β (+4) splice variant and the postsynaptic GluA1-4 subunits to physically force excitatory synapse assembly [173].

In vivo application of CPTX to the 5xFAD mouse model of Alzheimer’s disease successfully restored dendritic spine density and rescued impaired Schaffer collateral LTP. This structural and electrophysiological restoration translated directly to behavioral recovery, significantly improving spatial learning and contextual memory in the 5xFAD mice [173]. The therapeutic versatility of these molecular bridges extends to severe physical trauma; in spinal cord injury (SCI) models, CPTX promoted the de novo formation of GluA4-positive excitatory synapses within spared tissue, driving a sustained recovery of motor locomotion for over eight weeks post-injury [173].

### 7.5. Rescuing Local Translation: Modulating mTOR and Proteostasis Pathways for Synaptic Recovery

The collapse of localized translation and proteostasis is a defining signature of neurodegeneration, prompting interventions that modulate the mammalian target of rapamycin (mTOR) signaling pathway [174]. In the Tg2576 Alzheimer’s mouse model, Aβ oligomers impair the activity-dependent upregulation of mTOR, culminating in severe deficits in protein synthesis-dependent LTP [175]. Pharmacological disinhibition of mTOR using GSK3 antagonists, such as kenpaullone or LiCl, releases the inhibitory constraint of the TSC2 complex, thereby restoring long-lasting LTP in these pathological networks [175]. The profound impact of mTOR activation on structural plasticity is further corroborated by the mechanism of rapid-acting glutamatergic antidepressants (e.g., ketamine), which trigger the mTOR-dependent local translation of critical scaffolding proteins like PSD-95 and AMPA subunits like GluA1 [176,177,178].

Beyond the mTOR cascade, the unfolded protein response (UPR) and the eukaryotic initiation factor 2α (eIF2α) pathways represent critical targets for restoring synaptic proteostasis. The accumulation of misfolded proteins induces the hyperphosphorylation of eIF2α, which globally arrests cap-dependent translation and starves the synapse of plasticity-related proteins [179]. Pharmacological suppression of the eIF2α kinase PERK using the small molecule inhibitor GSK2606414 successfully restores local translation, preventing tau-mediated neuropathology, rescuing behavioral defects, and alleviating the aberrant long-term depression (LTD) characteristic of AD models [180]. Furthermore, restoring the bioenergetic capacity of spatially stable dendritic mitochondria is imperative to fuel this local translation and preserve synaptic resilience as shown in Figure 3.

### 7.6. Limitations and Translational Challenges

The strategies described above share a set of limitations that a balanced review should state explicitly. The most instructive is the clinical experience with nerinetide. Despite an unusually strong preclinical record, the ESCAPE-NA1 trial, which randomized 1105 patients with acute ischaemic stroke undergoing endovascular thrombectomy, found no difference between nerinetide and placebo in the proportion of patients achieving a modified Rankin Scale score of 0–2 at 90 days (61.4% versus 59.2%; adjusted risk ratio 1.04, 95% CI 0.96–1.14; *p* = 0.35) [181]. Evidence of treatment-effect modification indicated that concomitant alteplase, which cleaves the peptide, inhibited the effect. The general point is important for the whole of this section: the pharmacology of an interfering peptide is highly sensitive to the biological context in which it is given, and a mechanism that is valid at the level of the synapse may not survive translation to a heterogeneous clinical population.

Delivery is the second constraint. TAT-based cell-penetrating peptides cross the blood–brain barrier inefficiently and non-selectively, are rapidly degraded by serum proteases and enter every cell type they encounter, so that the anatomical precision implied by the term precision synaptopharmacology is not in fact achieved at the level of biodistribution. The cationic, arginine-rich sequences that confer penetration also carry a risk of membrane destabilization and of dose-limiting toxicity. Monoclonal antibodies directed against C1q face the complementary problem, since brain penetration of systemically administered IgG is of the order of 0.1% of the plasma concentration, while sustained peripheral complement inhibition raises the possibility of infectious complications. Synthetic organizers such as CPTX must be delivered by direct infusion or viral vector, which restricts their application to focal, anatomically defined lesions.

Third, the mechanistic foundations are less secure than the therapeutic framework may suggest. Condensate biology has been characterized largely in reconstituted systems, often at protein concentrations and in ionic conditions that do not reproduce the crowded synaptic environment, and it remains unclear which of the LLPS phenomena observed in vitro are operative in the intact human brain. Fourth, and most importantly, essentially all of the structural rescue data reviewed here derive from transgenic rodent models that reproduce a defined proteinopathy in a young and otherwise healthy nervous system, whereas human disease develops over decades in an ageing brain with mixed pathology, vascular comorbidity and substantial genetic heterogeneity. The repeated failure of neuroprotective strategies that had succeeded in such models, of which nerinetide is only the most recent example, argues for a cautious appraisal of the timelines involved [181].

### 7.7. Bridging Neurorehabilitation and Precision Synaptopharmacology

The integration of neurorehabilitation with precision synaptopharmacology represents a paradigm shift toward active circuit engineering, moving beyond mere functional compensation to achieve structural regeneration. By utilizing molecular “structural enablers,” this approach provides the necessary architectural foundation for activity-dependent plasticity to translate into stable, long-term functional recovery. Central to this synergy is the deployment of synthetic extracellular scaffolding proteins like CPTX, which acts as a bidirectional trans-synaptic bridge to physically force the assembly of functional glutamatergic circuits. Building on work identifying synapse-organizing molecules such as FGF22, gene-therapy and biomaterial interventions can enhance de novo excitatory synapse formation and promote sustained motor recovery in severe spinal cord injury models [182,183,184]. Normalizing the molecular conditions for LTP is likely important for intensive rehabilitation. Work from many scientists shows that TAT-based interfering peptides can selectively disrupt pathological PSD-95–centered complexes (e.g., GluN2A/PSD-95/Rabphilin-3A or GluN2B/PSD-95/nNOS), thereby restoring more physiological NMDA receptor signaling and potentially creating a synaptic environment more responsive to training-induced activity [37,163,185]. Finally, the success of these strategies relies heavily on the bioenergetic and proteostatic health of the synapse. Furthermore, localized protein synthesis and coordination of mTOR-dependent translation with the stabilization of dendritic mitochondria by VAP-dependent tethering can provide local ATP to support the protein synthesis–and actin-dependent structural spine changes engaged by repetitive rehabilitative training [67,68,186,187]. Ultimately, this synergy offers a blueprint for regenerative systems biology, where molecular codes are used to direct the rebuilding of cognitive and motor circuits in the diseased or injured brain.

## 8. Methodology

This study follows a narrative review design aimed at synthesizing contemporary insights into the molecular architecture of synaptic diversity and its critical role in selective neuronal vulnerability. The analysis specifically focuses on how distinct protein interactomes and structural scaffolds within the post-synaptic density (PSD) and the presynaptic active zone dictate the relative resilience or susceptibility of neural circuits in Alzheimer’s Disease (AD) and Parkinson’s Disease (PD). By mapping these molecular “codes,” the review explores why specific synaptic populations are early targets for neurodegeneration while others maintain functional integrity. Consistent with this comparative aim, Alzheimer’s disease and Parkinson’s disease are treated in parallel and, following revision, at comparable depth; where the underlying evidence base is asymmetric, this is stated explicitly in the text rather than resolved by omission.

To ensure the quality and reproducibility of the narrative synthesis, a comprehensive literature search was conducted using PubMed, Google Scholar, and PubMed Central (PMC). The search was limited to English-language studies, prioritizing high-resolution molecular investigations such as those utilizing mass spectrometry-based proteomics, proximity labeling, and single-nucleus transcriptomics. This timeframe ensures the inclusion of the latest advancements in “synaptome” mapping and the integration of spatial omics data into the understanding of disease progression.

Thematic search strategies were implemented using specific Boolean operators (Booleans) to align with each section of the review. For the introductory assessment of diversity, the strings used included (“synaptic diversity” OR “synaptome”) AND (“molecular identity” OR “cell adhesion molecules”) AND “brain regions”. The investigation of the structural architecture of the PSD relied on (“postsynaptic density” OR “PSD”) AND (“scaffold proteins” OR “hub proteins”) AND (“structural stability” OR “protein interactome”). Maintenance mechanisms and local proteostasis were explored through (“synaptic local translation” OR “mRNA translation”) AND (“proteostasis” OR “phase separation”) AND “homeostasis”. Pathological vulnerability in AD was mapped using (“Alzheimer’s disease”) AND (“synaptic vulnerability” OR “selective vulnerability”) AND (“complement C1q” OR “microglial pruning”), while PD-specific dysfunction was searched with (“Parkinson’s disease”) AND (“synaptic failure” OR “alpha-synuclein interactome”) AND (“PSD reorganization” OR “striatal failure”). Methodological advancements were captured via (“Mass Synaptometry” OR “CyTOF” OR “proximity labeling”) AND “synaptome mapping” AND “proteomics”. Finally, the pharmacological and concluding sections were built upon (“interfering peptides” OR “small molecules”) AND (“synaptic stabilization” OR “protein–protein interaction”) AND “therapeutic targeting” and (“synaptic resilience” OR “resilient synapses”) AND (“molecular signatures” OR “biomarkers”) AND “neurodegeneration”. For the sections added during revision, the following additional strings were used: (“tetrapartite synapse” OR “perineuronal net”) AND “extracellular matrix” AND (“astrocyte” OR “microglia”); (“liquid–liquid phase separation” OR “biomolecular condensate”) AND (“astrocyte” OR “microglia” OR “neuroinflammation”); (“LRRK2” OR “GBA1” OR “Rab”) AND “synaptic dysfunction” AND “Parkinson’s disease”; (“blood–brain barrier” OR “pericyte”) AND (“synaptic” OR “cognitive decline”) AND (“Alzheimer’s disease” OR “Parkinson’s disease”); “platelets” AND (“amyloid precursor protein” OR “alpha-synuclein”) AND “biomarker”; and (“nerinetide” OR “cell-penetrating peptide”) AND (“randomised controlled trial” OR “blood–brain barrier delivery”). Every reference added during revision was verified against its PubMed record, and both the PMID and the DOI were checked for each entry.

Papers were selected via a purposive sampling approach to ensure direct relevance to the molecular aims of the study. To maintain experimental grounding and avoid circular reporting, data extraction was restricted exclusively to the body text, results, and discussion sections of the primary papers, intentionally ignoring bibliographical lists. A strict single-source citation rule was applied to each claim to maximize the diversity of evidence and provide a broader perspective on the tetrapartite synapse and neuroimmune interactions. This methodological rigor ensures that the resulting narrative accurately reflects the primary data regarding protein–protein interaction (PPI) and cellular crosstalk in the aging and diseased brain.

All figures shown in this paper were made using BioRender software (Created in https://BioRender.com).

## 9. Conclusions and Future Perspectives

The historical conceptualization of the synapse as a generic, monolithic relay station has been substantially revised. As outlined in this review, the transition toward a systems-level understanding of the “synaptome” reveals an unprecedented landscape of molecular diversity, governed by highly specific protein–protein interactomes, spatial compartmentalization, and localized proteostasis. This paradigm shift is not merely structural; it is fundamentally redefining our approach to neurodegenerative diseases.

The evidence underscores that synaptic vulnerability in conditions such as Alzheimer’s and Parkinson’s diseases is neither random nor uniform. Instead, it is inextricably linked to the native proteomic architecture of specific synaptic populations. Pathological oligomers—whether Aβ, hyperphosphorylated Tau, or mutant α-Synuclein—exploit this diversity by selectively hijacking critical functional hubs. By disrupting liquid–liquid phase separation (LLPS) dynamics, fragmenting lipid rafts, or mechanically uncoupling essential scaffold nodes like PSD-95 and Rabphilin-3A, these toxic interactomes trigger cascading failures in receptor trafficking and local energy grids, ultimately culminating in complement-mediated synaptic pruning.

Looking forward, the successful translation of synaptome mapping into clinical efficacy relies on the development of “precision synaptopharmacology.” Broad-spectrum receptor antagonists have consistently failed because they disrupt physiological transmission alongside pathological overactivation. A more promising direction appears to be structurally targeted intervention capable of fine-tuning the synapse, although, as set out in Section 7.6, none of the approaches below have yet demonstrated clinical benefit. Strategies such as interfering cell-penetrating peptides (e.g., NA-1 and TAT-based modulators) that uncouple aberrant postsynaptic complexes, monoclonal antibodies targeting the neuroimmune C1q tagging system, and the deployment of synthetic synaptic organizers (e.g., CPTX) represent the first wave of this revolution.

Future research must prioritize the integration of high-dimensional spatial transcriptomics with in vivo proximity labeling to dynamically track synaptic interactomes across the temporal continuum of disease progression. Furthermore, understanding the precise mechanisms by which localized translation and mitochondrial bioenergetics can be therapeutically rescued will be paramount. Ultimately, mapping the molecular barcodes of synaptic diversity will not only unravel the etiology of selective vulnerability but will provide the definitive blueprints for preserving and actively rebuilding cognitive circuits in the aging brain.

Four specific directions follow from this analysis. First, single-synapse multiomics, in which mass synaptometry, proximity labeling and spatial transcriptomics are applied to the same anatomically defined population, would allow the molecular signature of a vulnerable synapse to be measured rather than inferred from separate datasets. Second, longitudinal human imaging offers a complementary route: radioligands for synaptic vesicle glycoprotein 2A such as UCB-J already permit synaptic density to be quantified in vivo and have been shown to detect treatment-related changes in preclinical models, and their extension to interventional trials would supply the synaptic endpoint that this field presently lacks [188]. Third, machine-learning approaches applied to high-dimensional synaptome and condensate imaging data are already resolving phenotypes that conventional analysis does not separate [155], and comparable tools will be required to reduce single-synapse datasets to interpretable classes of vulnerability. Fourth, the genetic heterogeneity of both disorders argues for a personalized synaptopharmacology, in which the choice between scaffold-directed, neuroimmune and phase-directed intervention is guided by the risk-variant profile of the individual patient, for example by the presence of TREM2, APOE, LRRK2 or GBA1 variants that determine which of the mechanisms reviewed here is likely to predominate [145,147]. Progress on all four fronts will depend on the availability of human tissue and human cellular models, since the limitations of transgenic systems discussed in Section 7.6 are unlikely to be overcome by refinement of those systems alone.

## Data Availability

No new data were created or analyzed in this study. Data sharing is not applicable to this article.

## Abbreviations

The following abbreviations are used in this manuscript:

AD	Alzheimer’s Disease
PD	Parkinson’s Disease
LLPS	Liquid–liquid phase separation
SSD	Subsynaptic domain
PSD	Post-Synaptic Density
PPI	Protein–protein interaction
CAMs	Cell adhesion molecules
PTMs	Post-translational modifications
FASS	Fluorescence-Activated Synaptosome Sorting
CyTOF	Cytometry by time-of-flight mass spectrometry
ST	Spatial transcriptomics
snRNA-seq	Single-nucleus RNA sequencing
SV	Synaptic vesicle
LTP	Long-term potentiation
LTD	Long-term depression
mTOR	Mammalian target of rapamycin
ESPs	Extracellular scaffolding proteins
UPR	Unfolded protein response
VAP	Vesicle-associated membrane protein-associated protein
SCI	Spinal cord injury
